# Supplementary material for: Cohort profile: The Social media, smartphone use and Self-harm in Young People (3S-YP) study–A prospective, observational cohort study of young people in contact with mental health services
Source: PLoS One. 2024 May 22;19(5):e0299059. doi: 10.1371/journal.pone.0299059 (PMC11111019; doi:10.1371/journal.pone.0299059)
Supplement: S1 Table — (DOCX) [file pone.0299059.s001.docx]

**S1 Table. Outline of data collection schedule for the 6-month follow-up period**

| **Measure** | **Data collected at:** | | | | | | |
| --- | --- | --- | --- | --- | --- | --- | --- |
|  | **Baseline** | **Month 1** | **Month 2** | **Month 3** | **Month 4** | **Month 5** | **Month 6** |
| **Socio-**  **demographic questionnaire** | X |  |  |  |  |  |  |
| **Child and Adolescent Self-harm in Europe (CASE) Study criteria** | X | X | X | X | X | X | X |
| **Self-reported social media use** | X |  |  |  |  |  | X |
| **Self-reported smartphone use** | X |  |  |  |  |  | X |
| **Smartphone Addiction Scale - Short Version (SAS-SV)** | X |  |  |  | X |  | X |
| **Generalised Anxiety Disorder (GAD-7)** | X |  |  |  | X |  | X |
| **Patient Heath Questionnaire (PHQ-9)** | X | X |  | X |  | X | X |
| **PROMIS Sleep Disturbance Short Form / Pediatric Sleep Disturbance Short Form** | X |  | X |  | X |  | X |
| **Three-Item Loneliness Scale** | X |  |  | X |  |  | X |
| **Eight-Item Bullying Checklist** | X |  | X |  |  | X | X |
| **Exposure to Covid-19 questionnaire** | X |  |  |  |  |  | X |
| **Social media data upload** | X |  |  | X |  |  | X |
| **Smartphone metadata extraction** | X | X | X | X | X | X | X |
| **Electronic health records data extraction** | X |  |  |  |  |  | X |
