# Supplementary material for: Cohort profile: The Social media, smartphone use and Self-harm in Young People (3S-YP) study–A prospective, observational cohort study of young people in contact with mental health services
Source: PLoS One. 2024 May 22;19(5):e0299059. doi: 10.1371/journal.pone.0299059 (PMC11111019; doi:10.1371/journal.pone.0299059)
Supplement: S2 Table — (DOCX) [file pone.0299059.s002.docx]

**S2 Table. Characteristics of approached population and enrolments from EHR data at screening**

| **Variable**  n (%) | | **Approached population** | **Did not enrol** | **Enrolled** | **3S-YP study cohort** |
| --- | --- | --- | --- | --- | --- |
|  | | N = 1,288 | N = 900 | N = 388 | N = 362 |
| **Age category** | |  |  |  |  |
|  | 13-15 | 163 (12.7) | 118 (13.1) | 45 (11.6) | 44 (12.2) |
|  | 16-17 | 291 (22.6) | 200 (22.2) | 91 (23.5) | 85 (23.5) |
|  | 18-21 | 514 (40.0) | 353 (39.2) | 161 (41.5) | 150 (41.4) |
|  | 22-25 | 320 (24.8) | 229 (25.4) | 91 (23.5) | 83 (22.9) |
|  | Missing | - | - | - | **-** |
| **Gender** | |  |  |  |  |
|  | Female | 790 (61.3) | 519 (57.7) | 271 (70.0) | 254 (70.2) |
|  | Male | 477 (37.0) | 369 (41.0) | 108 (27.8) | 100 (27.6) |
|  | Other/missing | 21 (1.6) | 12 (1.3) | 9 (2.3) | 8 (2.2) |
| **Ethnicity** | |  |  |  |  |
|  | Any White background | 721 (56.0) | 493 (54.8) | 228 (58.8) | 213 (58.8) |
|  | Any Black or Black British background | 219 (17.0) | 164 (18.2) | 55 (14.2) | 51 (14.1) |
|  | Any Mixed or Multiple ethnic background | 152 (11.8) | 106 (11.8) | 46 (11.9) | 42 (11.6) |
|  | Any Asian or Asian British background | 56 (4.4) | 36 (4.0) | 20 (5.2) | 19 (5.2) |
|  | Any other background | 38 (3.0) | 27 (3.0) | 11 (2.8) | 11 (3.0) |
|  | Missing | 102 (7.9) | 74 (8.2) | 28 (7.2) | 26 (7.2) |
| **Primary diagnosis** | |  |  |  |  |
|  | Mood (affective) disorders  (F30-39) | 109 (8.5) | 71 (7.9) | 38 (9.8) | 34 (9.4) |
|  | Anxiety, dissociative, stress-related, somatoform and other nonpsychotic mental disorders  (F40-48) | 353 (27.4) | 240 (26.7) | 113 (29.1) | 106 (29.3) |
|  | Behavioral syndromes associated with physiological disturbances and physical factors  (F50-59) | 118 (9.2) | 73 (8.1) | 45 (11.6) | 43 (11.9) |
|  | Disorders of adult personality and behavior  (F60-69) | 99 (7.7) | 61 (6.8) | 38 (9.8) | 36 (9.9) |
|  | Pervasive and specific developmental disorders (F84) | 111 (8.6) | 82 (9.1) | 29 (7.5) | 26 (7.2) |
|  | Behavioral and emotional disorders with onset usually occurring in childhood and adolescence (F90-98) | 219 (17.0) | 160 (17.8) | 59 (15.2) | 58 (16.0) |
|  | Unspecified mental disorder (F99) | 80 (6.2) | 62 (6.9) | 18 (4.6) | 18 (5.0) |
|  | Other  (All F codes below 30/all Z codes/  X78) | 115 (8.9) | 86 (6.7) | 29 (7.5) | 25 (19.4) |
|  | Missing | 84 (6.5) | 65 (7.2) | 19 (4.9) | 16 (4.4) |
