# Supplementary material for: Cohort profile: The Social media, smartphone use and Self-harm in Young People (3S-YP) study–A prospective, observational cohort study of young people in contact with mental health services
Source: PLoS One. 2024 May 22;19(5):e0299059. doi: 10.1371/journal.pone.0299059 (PMC11111019; doi:10.1371/journal.pone.0299059)
Supplement: S3 Table — (DOCX) [file pone.0299059.s003.docx]

**S3 Table. Data availability for self-reported measures from baseline and follow up assessments for the total cohort**

| **Measure** | **Data collected at:** | | | | | | | | | | | | | |
| --- | --- | --- | --- | --- | --- | --- | --- | --- | --- | --- | --- | --- | --- | --- |
|  | **Baseline**  (N = 362) | | **Month 1**  (N = 360)^a^ | | **Month 2**  (N = 359) | | **Month 3**  (N = 359) | | **Month 4**  (N = 355) | | **Month 5**  (N = 353) | | **Month 6**  (N = 351) | |
|  | n | missing | n | missing | n | missing | n | missing | n | missing | n | missing | n | missing |
| **Completed** | 362 | 0 | 189 | 85 | 219 | 140 | 185 | 174 | 162 | 193 | 146 | 207 | 230 | 121 |
| **Socio-demographics** | 362 | - | - | - | - | - | - | - | - | - | - | - | - | - |
| **Child and Adolescent Self-harm in Europe (CASE) Study criteria** | 358 | 4 | 183 | 91 | 215 | 144 | 181 | 178 | 161 | 194 | 144 | 209 | 225 | 126 |
| **Generalized Anxiety Disorder Scale (GAD-7)** | 360 | 2 | - | - | - | - | - | - | 158 | 197 | - | - | 210 | 141 |
| **Patient Health Questionnaire (PHQ-9)** | 360 | 2 | 180 | 94 | - | - | 178 | 181 | - | - | 142 | 211 | 210 | 141 |
| **PROMIS Pediatric Sleep Disturbance Short Form (for 5-17 yrs)^b^** | 119 | 1 | - | - | 70 | 47 | - | - | 48 | 68 | - | - | 74 | 41 |
| **PROMIS Sleep Disturbance Short Form**  **(for ≥18 yrs)** | 239 | 3 | - | - | 144 | 98 | - | - | 112 | 127 | - | - | 150 | 86 |
| **Eight-item bullying checklist** |  |  |  |  |  |  |  |  |  |  |  |  |  |  |
| Total score | 349 | 13 | - | - | 109 | 250 | - | - | - | - | 79 | 274 | 209 | 142 |
| Traditional bullying | 358 | 4 | - | - | 218 | 141 | - | - | - | - | 142 | 211 | 211 | 140 |
| Cyberbullying | 352 | 10 | - | - | 218 | 141 | - | - | - | - | 142 | 211 | 210 | 141 |
| **Three-Item Loneliness Scale** | 354 | 8 | - | - | - | - | 178 | 181 | - | - | - | - | 223 | 128 |
| **Self-reported social media use** | 361 | 1 | - | - | - | - | - | - | - | - | - | - | 217 | 134 |
| **Self-reported smartphone use** | 357 | 5 | - | - | - | - | - | - | - | - | - | - | 213 | 138 |
| **Smartphone Addiction Scale - Short Version (SAS-SV)** | 356 | 6 | - | - | - | - | - | - | 156 | 199 | - | - | 205 | 146 |

^a^Eighty-six young people who completed the baseline questionnaire within seven days of the next month, did not receive the month 1 questionnaire.

^b^Two young people had their 18^th^ birthday between being invited to participate and enrolment and received the Pediatric Sleep Disturbance Short Form at baseline and for the duration of the follow up period.
