# Supplementary material for: Cohort profile: The Social media, smartphone use and Self-harm in Young People (3S-YP) study–A prospective, observational cohort study of young people in contact with mental health services
Source: PLoS One. 2024 May 22;19(5):e0299059. doi: 10.1371/journal.pone.0299059 (PMC11111019; doi:10.1371/journal.pone.0299059)
Supplement: S2 File — (DOCX) [file pone.0299059.s005.docx]

**S2 File. Detailed description of measures**

The following data were collected at baseline and follow up according to the schedule presented in S1 Table. Detail on the measurements is provided below. For this publication we summarise information collected at baseline and data availability over the 6-month follow-up period. More detail on current self-harm, social media uploads, smartphone metadata and interview data will be included in future publications.

Primary outcome

*Self-reported self-harm*

Self-reported history of and current self-harm were assessed using the Child and Adolescent Self-harm in Europe (CASE) Study criteria [1] at baseline and each monthly follow-up. This measure comprises two items for assessing the presence and type of self-harm. The first item captured the presence of prior self-harm - “Have you ever deliberately taken an overdose (e.g. of pills or other medication) or tried to harm yourself in some other way (such as cut yourself)?”, with response options of “no”, “yes, once” and “yes, more than once”. Additional items were included at baseline to capture age (in years) when the individual first self-harmed and last self-harmed prior to baseline. The first item was modified for monthly questionnaires to capture self-harm in the past month, followed by a calendar for respondents to select the date(s) in the past month when self-harm occurred. Responses to the second item - “Describe what you did to yourself on that occasion. Please give as much detail as you can – for example, the name of the drug taken in an overdose” – were used to code the type of self-harm. To minimise potential participant burden, the monthly questionnaire was not adapted to collect information on the type of self-harm for each date selected, so types of self-harm indicated in the free text response applied to any dates selected. AB coded types of self-harm from the free-text responses with any discrepancies discussed at consensus with RD. We only considered this measure as missing if all the items were missing.

*Clinician-recorded self-harm*

Clinician-recorded history of self-harm (i.e., occurring prior to the baseline assessment) was identified through manual inspection of risk assessment forms in the EHR of consenting participants using ePJS. For participants with several risk assessment forms, the most recent risk assessment form prior to baseline was inspected and only where no self-harm was described, were further forms inspected. Information from free text and structured fields was used to determine the presence of prior self-harm as a dichotomous outcome. Information from free text fields was used to code the type(s) of self-harm. This data was only considered missing for consenting participants where no risk assessment form was available.

Using a similar approach to research by Polling et al. [2,3], clinician-recorded current self-harm was identified using the CRIS system to extract all free text entries that contained any self-harm-related keywords between baseline and month 6 for consenting participants. Data was extracted from all document types, aside from risk assessment forms that ceased being updated in the CRIS system during the study period. Keywords previously used [2,3] were supplemented with others identified through piloting the data extraction and coding process. Extracted data was re-identified and full entries were manually inspected using ePJS. To ensure there was no duplication of events, entries were inspected in chronological order and only one entry was coded for each event - selected according to the depth of information on timing and type of self-harm. Entries were coded according to the presence of self-harm, presence of self-harm during the participation period, date when self-harm occurred, and type of self-harm. Following which, all risk assessment forms completed during the participation period were manually inspected to detect any new events not already coded.

Researchers were trained by AB and RD in the data extraction and coding process. Data extraction and coding of the presence of self-harm was conducted by at least one researcher (AB, GW and AVV), with AB validating all self-harm events and coding date and type of self-harm. Any discrepancies or uncertainties were discussed in consensus meetings with RD, and through further discussion with Polling, another clinical academic field expert.

*Classification of self-harm*

Self-harm was classified in accordance with the National Institute of Health and Care Excellence definition: “intentional self-poisoning or -injury carried out by an individual, irrespective of the apparent purpose of the act” [26] and previous research outlining clinically accepted forms of self-harm. [2,3] Clinically defined self-harm comprised of (1) self-poisoning, (2) self-injury, (3) both self-poisoning and self-injury, and (4) other types of self-harm. Self-poisoning included any events where an individual consumed more than the recommended dose of a non-recreational drug, a poisonous amount of a recreational drug if it was a discrete event, or a substance not intended for human consumption, with the intent to self-harm identified by the individual or a clinician. Self-injury included any events involving intentional self-inflicted injury (e.g., self-cutting, biting, burning), regardless of how superficial the damage was. It did not include descriptions of habitual or repetitive self-injurious behaviours. Both self-poisoning and self-injury included any events where self-poisoning and self-injury took place during the same episode. Other types of self-harm included any events involving other forms of self-harm (e.g., attempted strangulation, running into traffic, jumping from a height), regardless of whether an injury was sustained, that occurred in isolation or in combination with self-poisoning and/or self-injurious behaviours.

We also employed a broader, more inclusive definition including behaviours not traditionally considered as self-harm where there was a stated intention to self-harm. The decision to include this broader definition was motivated by a desire to capture events described as self-harm that would otherwise have been omitted had we solely employed a clinical definition. The broader definition of self-harm similarly comprised of (1) self-poisoning, (2) self-injury, and (3) both self-poisoning and self-injury, however (4) other types of self-harm included any events involving alcohol poisoning, descriptions of other prolonged substance misuse (as distinct from a discrete self-poisoning event), and disordered eating behaviours (e.g. fasting, excessive exercise) if there was a stated intention to self-harm. We did not include any of these additional behaviours if there was not a stated intent to self-harm. In this publication we only present data on the presence of clinically and broadly defined history of self-harm, further detail on the types of self-harm will be included in future publications.

*Determining timing of current self-harm*

To ensure we did not duplicate current self-harm events, self-report and clinician-recorded prospective data were merged. Information on how we managed current self-harm events where the date of an event was not specified in the self-report or clinician-recorded data, where it was not a discrete event, or where there was overlap between self-report and clinician-recorded data, will be included in future publications presenting prospective data.

Secondary outcomes

*Anxiety symptoms*

Symptoms of anxiety were assessed using the Generalized Anxiety Disorder Scale (GAD-7) [4] at baseline and months 4 and 6. This measure comprises seven items assessing symptoms of generalised anxiety disorder in the past two weeks. Items are rated on a four-point Likert scale. Responses to each item sum to generate a total score ranging between 0 and 21, with scores of 5, 10, and 15 representing the cut-off points for mild, moderate, and severe anxiety, respectively. If only 1 item was missing, it was imputed with the mean of the completed items, otherwise the total score was treated as missing.

*Depression symptoms*

Symptoms of depression were assessed using the Patient Health Questionnaire (PHQ-9) [5] at baseline and months 1, 3, 5 and 6. This is a nine-item measure assessing symptoms of depression in the past two weeks. Items are rated on a four-point Likert scale. Responses to each item sum to generate a total score ranging between 0 and 27, with scores of 5, 10, 15, and 20 representing the cut-off points for mild, moderate, moderately severe, and severe depression, respectively. If 2 or fewer items were missing, they were imputed with the mean of the completed items, otherwise the total score was treated as missing.

*Sleep disturbance symptoms*

Symptoms of sleep disturbance in 13–17-year-olds (age at time of approach) were assessed using the Paediatric Sleep Disturbance Short Form V1.0 4a [6] at baseline and months 2, 4 and 6. This measure consists of 4 items assessing self-reported perceptions of difficulty with falling asleep, staying asleep through the night, having trouble sleeping or a problem with sleep in the past 7 days. Items are rated on a 5-point Likert scale. Responses to each item sum to generate a total raw score ranging between 4 and 20 and standardized using a T-score metric, with T-scores of 56, 60 and 66 representing the cut-off points for mild, moderate, and severe sleep disturbance, respectively. If only 1 item was missing, it was imputed with the mean of the completed items prior to rescaling into a T-score, otherwise the total score was treated as missing.

Symptoms of sleep disturbance in adults (≥18 year olds) were assessed using the Patient-Reported Outcomes Measurement Information System (PROMIS) Sleep Disturbance Short Form V1.0 4a [7] at baseline and months 2, 4 and 6. This measure consists of 4 items assessing self-reported perceptions of sleep quality, refreshment and perceived difficulty with falling asleep or having problematic sleep in the past 7 days. Items are rated on a 5-point Likert scale. Responses to each item sum to generate a total raw score ranging between 4 and 20 and standardized using a T-score metric, with T-scores of 55, 60 and 70 representing cut-off points for mild, moderate, and severe sleep disturbance, respectively. If only 1 item was missing, it was imputed with the mean of the completed items prior to rescaling into a T-score, otherwise the total score was treated as missing.

*Bullying victimisation*

Bullying victimisation was assessed using the Eight-Item Bullying Checklist derived from the Revised Olweus Bully/Victim Questionnaire (R-OBVQ) [8,9] at baseline and months 2, 5 and 6. This measure consists of 8 items assessing the occurrence and frequency of several types of traditional and cyber bullying in the past couple of months. Items are rated on a five-point Likert scale. Responses to each item sum to generate a total score ranging between 8 and 40, with higher scores indicative of bullying victimisation. If only 1 item was missing, it was imputed with the mean of the completed items, otherwise the total score was treated as missing. Binary indicators of regular traditional and cyber bullying victimisation were calculated using a cut-off of three (“2 or 3 times a month”) or greater for any of items 1-6 and 7-8, respectively [9,10]. Traditional bullying was only considered missing if all of items 1-6 were missing and cyberbullying was only considered missing if both items 7 and 8 were missing.

*Loneliness*

Feelings of loneliness were assessed using the Three-Item Loneliness Scale [11] at baseline and months 3 and 6. This measure comprises three-items assessing three dimensions of loneliness: relational connectedness, social connectedness and self-perceived isolation. Items are rated on a three-point Likert scale. Responses to each item sum to generate a total score ranging between 3 and 9, with higher scores indicative of greater feelings of loneliness. If any items were missing, then the total score was treated as missing.

Exposures

*Self-report social media use*

Self-reported social media use was assessed using unvalidated items at baseline and month 6, including use of social media, most frequently used platform, main purpose of usage, average daily usage on weekdays and weekends, and latest time of weekday and weekend use. For example, “How much time do you usually spend on social media on weekends?”, with response options ranging between “Less than 30 minutes” and “More than 6 hours”. This measure was only considered missing if all the items were missing.

*Self-report smartphone use*

Self-reported smartphone use was assessed using unvalidated items at baseline and month 6, including most frequently used app, average daily usage on weekdays and weekends, latest time of weekday and weekend use, use of phone at mealtimes, phone in bedroom at night, nighttime power mode and self-evaluation of excessive use. For example, “How much time do you usually spend on your phone on weekends?”, with response options ranging between “Less than 30 minutes” and “More than 6 hours”. This measure was only considered missing if all the items were missing.

*Problematic smartphone use*

Problematic smartphone use was assessed using the Smartphone Addiction Scale-Short Version (SAS-SV) [12] at baseline and months 4 and 6. This measure comprised of 10 items assessing self-evaluation of smartphone addiction. Items are rated on a six-point Likert scale. Responses to each item sum to generate a total score ranging between 10 and 60, with a score of ≥31 demonstrating a sensitivity of 0.867 and specificity of 0.893 for smartphone addiction in adolescent males, while a score of ≥33 demonstrating a sensitivity of 0.875 and a specificity of 0.886 for smartphone addiction in adolescent females. There is no available published guidance on validated cut-offs for young people who do not identify as either male or female. In this study, for participants that prefer to self-describe, we applied the higher threshold of ≥33 to categorize excessive phone use. If 2 items or fewer were missing, we imputed the missing items with the mean response of the completed items, otherwise the total score was treated as missing.

*Social media uploads*

Social media meta-, imagery and textual data were obtained from social media data uploads from consenting participants. Data was uploaded from Facebook, Instagram, Snapchat, TikTok, Twitter and YouTube following baseline and months 3 and 6 questionnaires, with participants able to choose which platforms and accounts to share data from. Individuals most recent valid data uploads from each platform were processed. Outcomes of interest include duration of usage (hours/minutes per day), timing of usage (diurnal/nocturnal posting), frequency of usage (e.g. visits to platforms per day) and type of use. For the purposes of this publication, we only present processed data availability without consideration for data quality, further detail will be included in future publications of prospective data.

*Smartphone metadata*

Smartphone metadata was collected via the app for consenting participants (apart from iPhone users due to iOS data protection regulations). Data was extracted continuously from the point of installation using App Usage API’s. Outcomes of interest include intensity of usage by time of day, frequency and duration of incoming and outgoing telephone calls, frequency of incoming and outgoing text messages and length of time spent on apps. For this publication we only present data available during the participation period, not considering data quality, further detail will be included in future publications presenting prospective data.

Other measurements

*Sociodemographics*

Self-reported sociodemographic data was collected at baseline, including age, gender, ethnicity, education, employment, smoking, electronic cigarette use, alcohol consumption and drug use. Smoking, electronic cigarette use, alcohol consumption and drug use [13]were assessed using items from the NHS Digital survey of secondary school pupils in England in 2018. This measure was only considered missing if all the items were missing.

*Exposure to Covid-19*

Self-reported exposure to Covid-19 was measured at baseline and month 6 using items from The Centre for Longitudinal Studies (CLS) and the MRC Unit for Lifelong Health and Ageing (LHA) - Wave 2 of the COVID-19 Survey in Five National Longitudinal Studies. This measure was only considered missing if all of the items were missing.

*EHR extracted data*

Sociodemographic and clinical data were extracted from consenting participants EHR using the CRIS system following baseline and month 6 questionnaires. For this publication, we present data available prior to baseline including Indices of Multiple Deprivation (IMD), number of years since first accepted referral to SLaM, psychiatric inpatient admissions and sections under the Mental Health Act.

*Post-study participation interview*

The post-study participation interview topic guide was co-designed with young people to facilitate informal discussions on the experience of participation and perspectives on the research topic more broadly.

References

1. Madge N, Hewitt A, Hawton K, Wilde EJ de, Corcoran P, Fekete S, et al. Deliberate self-harm within an international community sample of young people: comparative findings from the Child &amp; Adolescent Self-harm in Europe (CASE) Study. Journal of Child Psychology and Psychiatry. 2008;49(6):667–77.

2. Polling C, Bakolis I, Hotopf M, Hatch SL. Differences in hospital admissions practices following self-harm and their influence on population-level comparisons of self-harm rates in South London: an observational study. BMJ Open. 2019 Oct 1;9(10).

3. Polling C, Tulloch A, Banerjee S, Cross S, Dutta R, Wood DM, et al. Using routine clinical and administrative data to produce a dataset of attendances at Emergency Departments following self-harm. BMC Emerg Med. 2015 Jul 16;15(1):1–8.

4. Spitzer RL, Kroenke K, Williams JBW, Löwe B. A Brief Measure for Assessing Generalized Anxiety Disorder. Arch Intern Med. 2006;166(10):1092.

5. Kroenke K, Spitzer RL, Williams JBW. The PHQ-9: Validity of a brief depression severity measure. J Gen Intern Med. 2001;16(9):606–13.

6. Forrest CB, Meltzer LJ, Marcus CL, De La Motte A, Kratchman A, Buysse DJ, et al. Development and validation of the PROMIS Pediatric Sleep Disturbance and Sleep-Related Impairment item banks. Sleep. 2018 Jun 1;41(6).

7. Yu L, Buysse DJ, Germain A, Moul DE, Stover A, Dodds NE, et al. Development of Short Forms From the PROMIS^TM^ Sleep Disturbance and Sleep-Related Impairment Item Banks. Behavioral Sleep Medicine. 2011;10(1):6–24.

8. Olweus D. Revised Olweus Bully/Victim Questionnaire. J Psychopathol Behav Assess. 1996;

9. Przybylski AK, Bowes L. Cyberbullying and adolescent well-being in England: a population-based cross-sectional study. Lancet Child Adolesc Health. 2017;1(1):19–26.

10. Cook CR, Williams KR, Guerra NG, Kim TE, Sadek S. Predictors of bullying and victimization in childhood and adolescence: A meta-analytic investigation. School Psychology Quarterly. 2010 Jun;25(2):65–83.

11. Hughes ME, Waite LJ, Hawkley LC, Cacioppo JT. A Short Scale for Measuring Loneliness in Large Surveys: Results From Two Population-Based Studies. Res Aging. 2004;26(6):655–72.

12. Kwon M, Kim DJ, Cho H, Yang S. The Smartphone Addiction Scale: Development and Validation of a Short Version for Adolescents. Choi DS, editor. PLoS One. 2013;8(12):e83558.

13. Smoking, Drinking and Drug Use among Young People in England 2018 [Internet]. 2018. Available from: https://digital.nhs.uk/data-and-information/publications/statistical/smoking-drinking-and-drug-use-among-young-people-in-england/2018#resources
